# Efficient and Scalable Object Localization in 3D on Mobile Device

**DOI:** 10.3390/jimaging8070188

**Published:** 2022-07-08

**Authors:** Neetika Gupta, Naimul Mefraz Khan

**Affiliations:** Department of Electrical, Computer & Biomedical Engineering, Toronto Metropolitan University, Toronto, ON M5B 2K3, Canada; n77khan@ryerson.ca

**Keywords:** object localization, object detection, ARCore

## Abstract

Two-Dimensional (2D) object detection has been an intensely discussed and researched field of computer vision. With numerous advancements made in the field over the years, we still need to identify a robust approach to efficiently conduct classification and localization of objects in our environment by just using our mobile devices. Moreover, 2D object detection limits the overall understanding of the detected object and does not provide any additional information in terms of its size and position in the real world. This work proposes an object localization solution in Three-Dimension (3D) for mobile devices using a novel approach. The proposed method works by combining a 2D object detection Convolutional Neural Network (CNN) model with Augmented Reality (AR) technologies to recognize objects in the environment and determine their real-world coordinates. We leverage the in-built Simultaneous Localization and Mapping (SLAM) capability of Google’s ARCore to detect planes and know the camera information for generating cuboid proposals from an object’s 2D bounding box. The proposed method is fast and efficient for identifying everyday objects in real-world space and, unlike mobile offloading techniques, the method is well designed to work with limited resources of a mobile device.

## 1. Introduction

Three-dimensional object detection has a wide variety of applications in self driving cars, environment mapping, augmented reality, etc. However, state-of-the-art approaches for 2D/3D object detection, discussed in Section 2, involve heavy computations that cannot be fully supported by the constrained hardware resources of a mobile device. In such a situation, we always have to identify a balanced trade-off between computational processing speed and accuracy.

Current 2D/3D object detection solutions for mobile devices might provide satisfactory results but often have limited scalability, e.g., (1) they are constrained for a preprocessed environment which requires significant preparatory effort; (2) tracking of the detected objects depends upon extracted features, e.g., high-gradient corners, across the image using external feature extractors; (3) they require large 3D annotated datasets for training and testing that demand significant amounts of continuous investment in human resources and time.

In the present research, we devised a scalable method to automate 2D object detection on a mobile device and localize objects in real-world coordinates. In this work, we propose an efficient framework to estimate the 3D bounding box of the detected object using a single RGB image where R, G and B respectively defines Red, Green, and Blue color components for each individual pixel of an image. The RGB image is captured through a mobile camera and is processed using ARCore [1] to identify additional information of the physical camera in the real-world space. We leverage ARCore functionalities to better understand the environment we are working with as it uses SLAM for localizing the device and continuously detects feature points and planes to enhance its understanding of the real world. Our method requires no 3D annotated dataset to configure and compute the real-world coordinates of the object. We eliminate this overhead by leveraging the already existing 2D annotated datasets.

**Figure 1:**
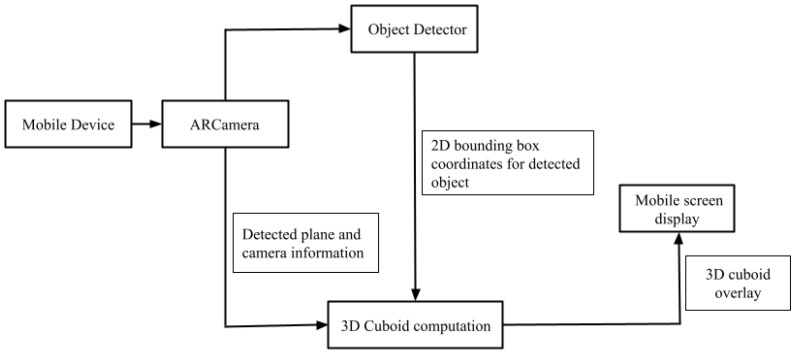
Flowgraph of the proposed method.

The following contributions are made in the proposed framework illustrated in Figure 1.

- Our work enables 2D object detection in a mobile device using a pretrained CNN model.
- Once the 2D bounding box for the detected object in the image scene is obtained, a 3D cuboid for the object is estimated using 2D bounding box coordinates and vanishing point sampling. ARCore is used to determine camera pose and rotation matrix for the vanishing point computations.
- Overall processing time is reduced by optimizing the number of generated 3D cuboid proposals using additional information from the horizontal planes detected using ARCore. The proposed framework works well with everyday objects.

## 2. Related Work

Two-dimensional object detection is an extensively researched field and over the years various methods have been developed, each exhibiting higher accuracy over the others either for a particular application with a specific set of objects or for different environment conditions. One such recent method for video saliency detection is proposed by Jian et al. [2]. However, deploying an object detection CNN model on a mobile device comes with its own set of challenges. It becomes difficult to balance accuracy and real-time capability on a resource-constrained platform. Real-time requirements in such cases are fulfilled either by offloading a part of computation from a mobile device to the cloud [3,4,5] or by shrinking the model down in size so that it fits and runs on computationally limited devices utilizing very low memory [6,7].

Mobile offloading is a familiar process but often struggles with drawbacks such as increased latency due to delayed network communication. This obstructs the overall experience of mobile continuous vision. Han et al. [3] applied Deep Neural Network (DNN) model optimization to produce variants of each model and processing time that schedule the model execution on a device and cloud to maximize accuracy while staying within resource bounds. The mobile device in this case is intermittently connected to the cloud. Ran et al. [4] proposed a deep learning framework called Deep Decision that provides a powerful server as a backend for the mobile device to allow the execution of deep learning models locally or remotely in the cloud. Liu et al. [5] employed a low latency offloading technique by separating the rendering and offloading pipeline and using a lightweight motion vector-based object tracking technique to maintain detection accuracy. However, they all suffer from long transmission latency and privacy concerns.

Apicharttrisorn et al. [8] in their work proposed a solution to perform object detection without offloading. Their proposed framework uses DNNs only when there is a need to detect new objects or reidentify objects that significantly change in appearance. Liu et al. [9] proposed a parallel detection and tracking pipeline to achieve real-time object detection performance without offloading. While running in parallel, the object detector and tracker switch among different model settings to consider the changing rate of video content.

There are many other advancements to minimize latency and computation time of deep learning models on mobile devices [10,11,12,13,14,15,16]. Lane et al. [10] provided distinct forms of resource optimization solutions for deep learning inference. Huynh et al. [11] proposed a way of running deep learning inference on mobile devices by offloading convolutional layers to mobile GPUs to speed up the processing. Xu et al. [14] accelerated the execution of CNN models by leveraging video temporal locality for continuous vision tasks. Fang et al. [15] dynamically designated an optimal resource and accuracy trade-off for a particular DNN model to fit its resource demand with respect to the available resources. Deep learning frameworks such as Caffe2 [17] and TensorFlow Lite [18] support direct execution of DNN models on mobile devices. These frameworks export pretrained models to perform inference on a mobile device. However, even with various optimizations, on-device inference using these frameworks is not fast enough as compared to running inference on powerful servers.

Three-dimensional object detection and environment understanding has become vital in the advancement of various computer vision applications today. State-of-the-art methods for computing 3D predictions of the detected object often require additional information and resources, for example, LiDAR sensors to collect spatial data [19,20,21,22]. Such solutions are not feasible when one is working with a mobile device. Google’s Objectron [23] performs 3D object detection on a mobile device in real time with Adreno 650 mobile GPU. However, the detection is limited to a finite number of objects, i.e., shoe, chair, cup and camera.

Current solutions to perform 2D/3D object detection on a mobile device provide satisfactory results but often have limited scalability and are constrained to a preprocessed environment which requires significant preparatory effort and resources. This work proposes an accurate and intelligent object localization solution for mobile devices using a novel approach by combining DNNs for 2D object detection with AR technology to recognize objects located in the environment and determine their real-world coordinates. Through our proposed work, we aim to provide an object-aware understanding of the surrounding environment while the user is being tracked through ARCore. An integrated object level understanding of the environment will enable AR/Virtual Reality (VR) solutions such as digital twins, metaverse, etc. to analyze and estimate the dynamic characteristics and real-time changes from physical space to virtual space.

## 3. Proposed Method

**Figure 2:**
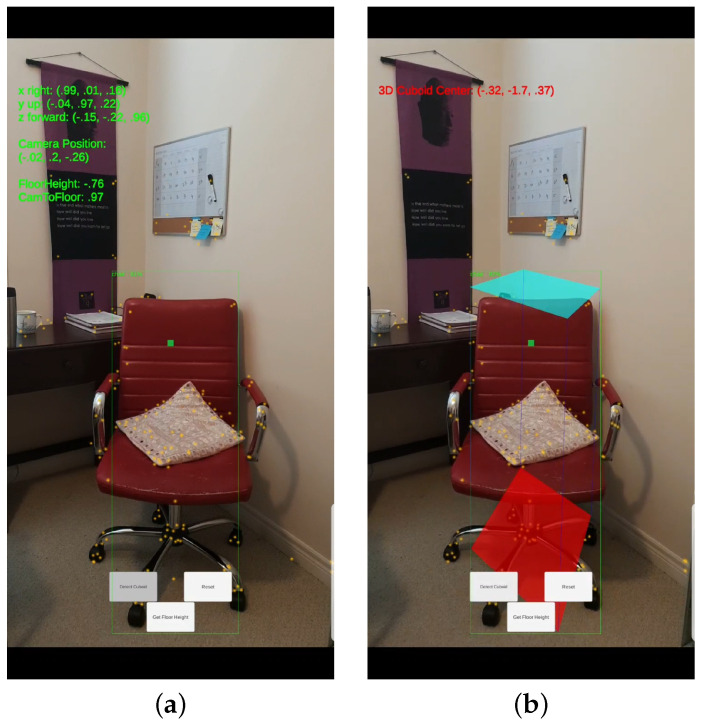
Screenshots of the mobile application developed based on our framework. (**a**) The 2D bounding box (green box) of the detected object using SSD-MobileNetV1, (**b**) the final 3D cuboid computed (light blue and red surfaces represent the top and bottom of the cuboid, respectively, and are joined with dark blue colored line segments) from the 2D bounding box.

Our objective is to develop a scalable solution for performing 3D object detection on a mobile device in order to localize objects in a real-world coordinate system. We developed an Android application using ARCore in the Unity3D engine for capturing RGB images and estimating the camera motion. The proposed framework of the mobile application is enabled on two button clicks. First button click, “Get Floor Height” (as shown in Figure 2), computes the height of the camera from the floor. The camera height is computed using ARCore’s capability to shoot a ray from the center of the screen. The center of the screen should be pointing towards the detected plane on the floor. Once the camera height is known, the app starts 2D object detection for the objects identified in the image scene. For a particular object of interest, the user can compute the 3D cuboid (3D bounding box) for the object by clicking the “Detect Cuboid” button. This computes the 3D world coordinates of the bounding box confining the object and coordinates of the cuboid center are visible on the screen.

### 3.1. 2D Object Detection

We used the TensorFlow Lite [18] framework to deploy a deep learning 2D object detection model on a mobile device. The model we used in our work is SSD-MobileNetV1 trained on the MS COCO dataset. MobileNets [24] are small effective deep learning models that are designed to meet the resource constraints of different use cases. These are the first known mobile computer vision models for TensorFlow that are designed to attain an efficient trade-off between accuracy and restricted resources of a mobile device application. In SSD-MobileNetV1, Single Shot Detector (SSD) [25] is used for performing object detection (localization) and classification, while MobileNetV1 is used as a feature extractor to perform detection. In our work, the camera feed is enabled using ARCore in order to obtain camera texture and light estimation information. By enabling ARCore while performing 2D object detection, we are able to take advantage of ARCore capabilities to better understand and estimate the environment we are working in.

### 3.2. 3D Cuboid Computation

Once we obtain the 2D bounding box for the detected object in the image scene, we estimate the 3D cuboid for the object using the method proposed by Yang and Scherer [26]. The 3D cuboid proposals are generated using two strong assumptions: (1) projected 3D cuboid corners should tightly fit the 2D bounding box, (2) objects are lying on the ground. Therefore, the world frame is built on the ground plane and hence the object’s roll/pitch angle becomes zero.

Now, a general 3D cuboid can be represented by 9 degree-of-freedom (DoF) parameters where 3 parameters define position *P*, 3 define rotation *R* and the last 3 define dimension *D*.

(1) $$P=\left[p_{x},p_{y},p_{z}\right];R=R\left(z,\alpha \right)R\left(y,\beta \right)R\left(x,\gamma \right);D=\left[d_{x},d_{y},d_{z}\right]$$

**Figure 3:**
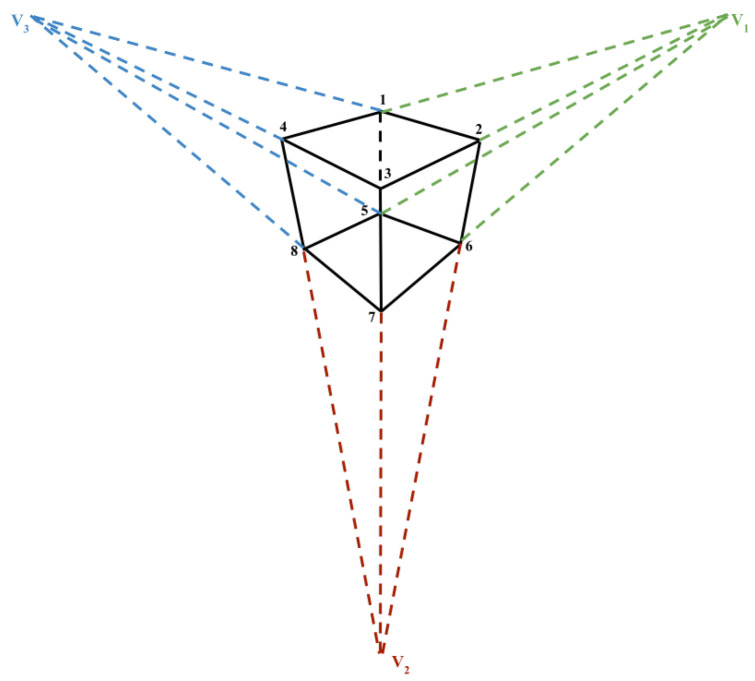
Vanishing point representation for a cube.

In the above equation, R(z,α), R(y,β), R(x,γ) represent the counterclockwise rotation through α angle about the *z* axis, β angle about the *y* axis and γ angle about the *x* axis, respectively. Making use of the assumption that projected corners of the cuboid should be confined within the 2D bounding box, there are only limited constraints that could be estimated with respect to the four sides of the bounding box. Therefore, vanishing points are used as additional information to estimate the 9 DoF parameters. A vanishing point is defined as a point on the image plane of a perspective drawing where the 2D perspective projections of mutually parallel lines in 3D space appear to converge (Figure 3). As represented in Figure 3, a cube is drawn using 12 edges. These 12 edges can be divided into 3 groups with each group containing 4 mutually parallel edges and every group potentially defines a vanishing point, giving us 3 vanishing points in total ($V_{1}$, $V_{2}$ and $V_{3}$).

**Figure 4:**
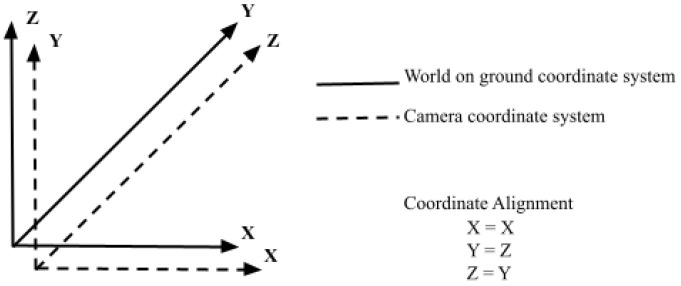
Alignment of world and camera coordinate system.

Therefore, as the 3D cuboid has three perpendicular axes, three vanishing points are known after projection. Their computation is based on the rotation matrix *R* with respect to camera frame and calibration matrix [26]. In our work, we use ARCore to determine camera pose and rotation matrix for computing the vanishing points. The transformation matrix from camera to world ground frame is determined by using the ARCamera pose. The coordinate systems followed for computation are represented in Figure 4 where the camera is defined as x right, y up, z forward and world ground frame is defined as x right, y forward, z upward. Additionally, following the assumption that the object is always placed on the ground, the camera will always be parallel to the ground. The scale of the object is determined by the camera height in the projection process. The camera height is calculated by shooting a ray from the center of the screen towards a detected horizontal plane on the floor managed by ARCore’s ARPlaneManager.

### 3.3. Optimization

We optimized the proposed framework to enable real-time processing for 3D cuboid computation. For one detected 2D bounding box, many 3D cuboid proposals are computed. These proposals are then ranked using a cost function stated in Equation (2) [26].

(2) $$E\left(C|I\right)=dist\left(C,I\right)+wt_{1}angle\left(C,I\right)+wt_{2}shape\left(O\right)$$

where the image is denoted as *I* and the cuboid proposal as *C*. Three primary costs considered are: (1) distance error (dist): measures the alignment of cuboid edges in 2D space with the image edges. Canny edge detector is used in this case to detect image edges. (2) Angle alignment error (angle): measures whether angles of long line segments align with vanishing points. (3) Shape error (shape): deals with the fact that similar 2D cuboid corners might generate quite different 3D cuboids. $wt_{1}$ and $wt_{2}$ are weight parameters set as $wt_{1}$ = 0.8, $wt_{2}$ = 1.5 [26]. In our work, line segments are detected using a Fast Line Detector (FLD) instead of using a Line Segment Detector (LSD) as used in [26]. The FLD is faster as compared to the LSD with no apparent performance degradation [27]. Therefore, we used the FLD for detecting line segments. We also modified the original approach of ranking the cuboid proposals by just using the cost function in Equation (2). In addition to it, we leverage the detected plane normal in 3D space (3Dnormal) and normal projected on and relative to screen space, i.e., in 2D space (screennormal), to minimize the number of cuboid proposals before applying the cost function.

- The dist and angle costs of the cost function are applied in the 2D image space. Therefore, before applying the 2 costs, we reduce the number of cuboid proposals using screennormal. We evaluate the angle made by the screen normal with the *x* axis in 2D image space ($\theta_{screen}$) using the following equation:
  (3) $$\theta_{screen/vp\_center}=atan2\left(y_{2d},x_{2d}\right)$$
  where atan2 is the four quadrant tangent inverse and point $\left(x_{2d},y_{2d}\right)$ represents the projected screen normal. We use the same equation (Equation (3)) to compute the angle of the vanishing point center projected in 2D image space ($\theta_{vp\_center}$). Next, we compute the difference between the two angles and, for a particular cuboid proposal to be selected for further processing, the value of angle difference should not exceed a given threshold. We set the threshold to 20° after experimenting with different values such as 45°, 30°, 20°, 15°.
- We further minimize the number of cuboid proposals using the 3Dnormal. The 3Dnormal is computed from the plane detected using ARCore. Direction angles computed for the 3Dnormal are α, β and γ which represent the angles formed by the normal with positive *x*, *y* and *z* axes, respectively, and are given as:
  (4) $$\alpha =\cos^{-1}\frac{x_{3d}}{mag\_P_{3d}};\beta =\cos^{-1}\frac{y_{3d}}{mag\_P_{3d}};\gamma =\cos^{-1}\frac{z_{3d}}{mag\_P_{3d}};$$
  where $\cos^{-1}$ is the cosine inverse, $P_{3d}\left(x_{3d},y_{3d},z_{3d}\right)$ represents the 3Dnormal and $mag\_P_{3d}$ represents the magnitude of the normal vector. We use the same equation (Equation (4)) to compute the direction angles made by a cuboid proposal with positive *x*, *y* and *z* axes. Next, the angle difference between respective direction angles is computed, which should not exceed a threshold. The threshold value in this case is also 20${}^{\circ }$ after experimenting with different values such as 25${}^{\circ }$, 20${}^{\circ }$, 18${}^{\circ }$, 15${}^{\circ }$, 10${}^{\circ }$. If for a particular cuboid proposal the angle difference value remains within the threshold, the cuboid is selected to be ranked according to the cost function as defined is Equation (2).

## 4. Experiments

The mobile application is deployed on a Samsung Galaxy S9 for all the experiments. In the proposed method, the user needs to start scanning the environment through the mobile app and, once the app is able to identify horizontal planes, the user clicks the button to compute height of the camera from the detected plane. After camera height computation, the app starts 2D object detection for the objects that are visible in the environment. The 3D cuboid computation is enabled as a button event in the mobile app. Once the framework is able to detect an object in the environment, 3D bounding box computation is triggered by clicking the corresponding button.

For better data acquisition and comparative analysis, experiments are carried out using a set of 27 images represented in Table 1. The images capture 9 different object categories, namely Book, Cellphone, Chair, Dog, Laptop, Mug, Potted_Plant, Table and Tennis_Racket at different orientations. All 27 images are captured using the same mobile device. Horizontal plane and camera information is obtained using ARCore. For each image, camera height is calculated using the approach discussed in Section 3.2 and the ground plane is defined as the surface on which the object is placed. The SSD-MobileNetV1 object detection model is used to localize the object in the image and generate a 2D bounding box. The model is trained on the MS COCO dataset which contains all the listed object categories for the captured images.

The proposed method is compared with [26] as it is an efficient method to generate high-quality 3D cuboid proposals from 2D bounding boxes using a single image. Table 2 tabulates the object label and confidence score for the object detected in respective images using SSD-MobileNetV1 and the corresponding 3D cuboids generated using [26] and our proposed method.

In the experiments, the SSD-MobileNetV1 object detector is used as an example for representing the entire workflow of the proposed method and to showcase the dependency of the 3D cuboid computation algorithm on the detected 2D bounding boxes. The efficiency of the 3D cuboid computation algorithm is also tested on the ground truth 2D bounding boxes for the objects and a few examples of the results are listed in Table 3. Since the results based on the ground truth are the best possible results for 2D object detection, no other 2D object detector is used for the comparative evaluation of the proposed framework.

Since 3D cuboid computation highly depends upon the accuracy of the 2D object detector, we noticed that in cases where SSD-MobileNetV1 is not able to detect the object in the image or the bounding box predicted by it is not correct, i.e., the entire object is not accurately confined within the 2D bounding box, the 3D cuboid generated by the framework is equally hindered. One such example is represented in Table 3. Table 3 tabulates results for 3 object categories, Mug, Table and Tennis_Racket. For the object category Table, there is no object detected by SSD-MobileNetV1 in the image and hence the 3D cuboid is not generated. However, when 2D bounding box coordinates are manually provided for the object, the proposed method is able to compute the 3D cuboid coordinates successfully.

Apart from the visual observations tabulated in Table 2 and Table 3, 3D-Intersection over Union (IoU) [28] is used as the evaluation metric. The ground truth cuboid for the objects in the images is created by manually drawing a cuboid on the object in the image space and the 2D image coordinates of the cuboid are converted to 3D camera coordinates using the Efficient Perspective-n-Point (EPnP) pose estimation algorithm [29]. The results are tabulated in Table 4 where the values show that our method always exhibits a comparatively equal or more accurate 3D cuboid for the detected object as compared to the state-of-the-art method [26].

**Figure 5:**
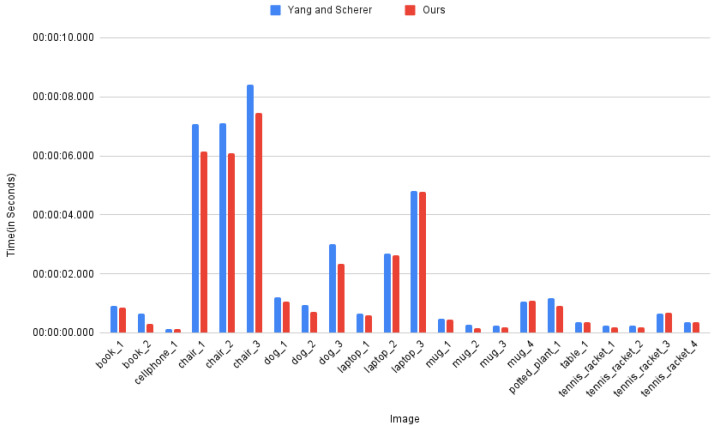
Comparison graph for time taken (in seconds) by [26] and our approach.

Figure 5 shows a comparison bar graph for the time taken (in seconds) by the two approaches. For some objects, the 2D detector failed to detect the object in the image and therefore they are not reported in the graph. One such example is the Cellphone object category where SSD-MobileNetV1 failed to detect the object in one of the images. The results presented in the bar graph show that our method is faster in comparison to [26].

## 5. Conclusions

In this paper, we propose a framework to localize objects in 3D using computationally constrained resources of a mobile device. The proposed solution determines the position of an object in real-world space and no depth information of the scene is required in order to compute the 3D coordinates of the detected object. A mobile application is developed using the proposed framework to facilitate data acquisition and continuous processing. Unlike mobile offloading techniques, our solution requires no external resources for computation and is therefore lightweight and scalable.

## Figures and Tables

**Table 1:** Images used for experiments.

Object Category	Image No.1	Image No.2	Image No.3	Image No.4
Book	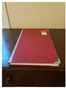	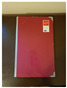		
Cellphone	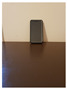	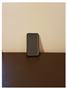		
Chair	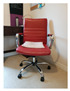	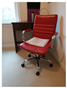	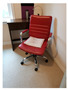	
Dog	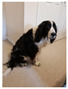	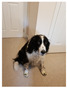	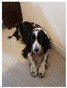	
Laptop	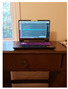	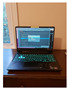	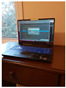	
Mug	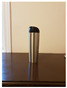	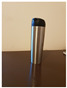	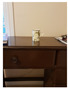	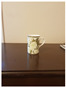
Potted_Plant	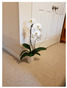	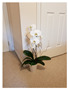	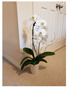	
Table	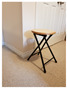	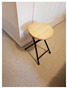	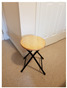	
Tennis_Racket	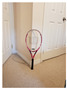	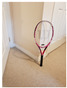	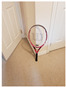	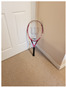

**Table 2:** Object predicted by SSD-MobileNetV1 (second column from the left) in the image and the corresponding 3D cuboid output using [26] and our approach.

Object Category	Object Predicted	Yang and Scherer [26]	Ours
Book	TV 56% (wrong prediction)	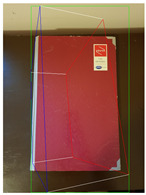	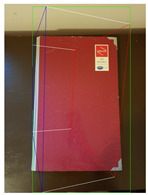
Chair	Chair 56% (correct prediction)	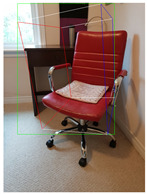	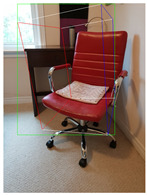
Dog	Dog 76% (correct prediction)	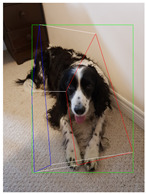	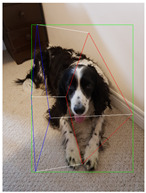
Potted_Plant	Potted Plant 53% (correct prediction)	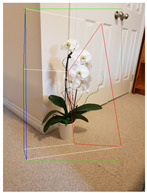	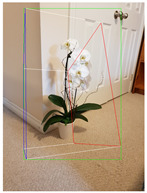

**Table 3:** 3D cuboid generated when 2D bounding box coordinates are obtained using SSD-MobileNetV1 and when defined manually. Note that in the case of object category Table, there is no object detected by SSD-MobileNetV1 and hence 3D cuboid is not generated.

Object Category	SSD-MobileNetV1		Manual	
	**2D Bounding Box**	**3D Cuboid**	**2D Bounding Box**	**3D Cuboid**
Mug	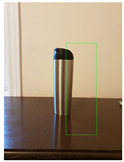	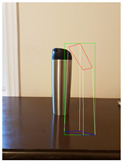	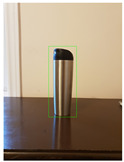	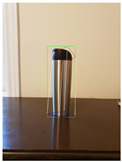
Table	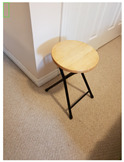	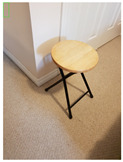	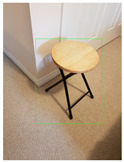	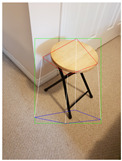
Tennis_Racket	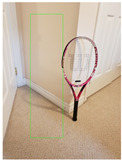	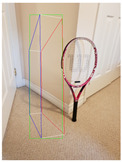	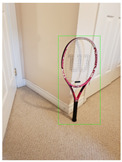	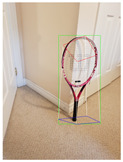

**Table 4:** The 3D-IoU results for generated cuboid by [26] and our approach.

Object Category	Book	Cellphone	Chair	Dog	Laptop	Mug	Potted Plant	Table	Tennis Racket
Yang and Scherer [26]	0.0903	0.0036	0.2804	0.0303	**0.1199**	**0.0248**	0.0993	**0.1934**	**0.0555**
Ours	**0.0989**	**0.0037**	**0.2806**	**0.0354**	0.1135	0.0238	**0.1188**	0.1847	0.0529

## Data Availability

Not applicable.
